# DHA and EPA levels in a piscivorous fish changed by preying upon stocked salmon fry

**DOI:** 10.1038/s41598-023-42530-2

**Published:** 2023-09-15

**Authors:** Koh Hasegawa, Yutaka Yano, Kentaro Honda, Yuhei Ogura

**Affiliations:** Fisheries Resources Institute, Japan Fisheries Research and Education Agency, Sapporo, Hokkaido 062-0922 Japan

**Keywords:** Ecology, Physiology, Zoology

## Abstract

Increases in prey population size can affect the physiology and ecology of upper-trophic level organisms. This phenomenon is known as a bottom-up effect. For example, the increased abundance of prey resources can trigger physiological (internal) changes in predators, such as improvements in nutritional status. However, these physiological aspects of bottom-up effects have not been considered. In this study, we tested the hypothesis that white-spotted charr *Salvelinus leucomaenis*, a salmonid fish, increases body stores of omega-3 fatty acids, especially docosahexaenoic acid (DHA) and eicosapentaenoic acid (EPA), by preying upon stocked hatchery-reared masu salmon *Oncorhynchus masou* fry in streams. The dynamics of fatty acid contents in charr inhabiting salmon-stocked and unstocked streams clearly support this hypothesis: fatty acid contents (DHA, EPA, and total fatty acid) increased after stocking in stocked streams, but not in unstocked streams. In addition, DHA increased with increasing body size of white-spotted charr and vice versa for EPA. The impacts of human activities, such as fish stocking, on freshwater ecosystems are a matter of serious concern for conservation. Future attempts to gain a comprehensive understanding of the impacts of fish stocking should consider not only community ecology but also physiology.

## Introduction

Bottom-up effects are benefits accrued by upper-trophic level organisms because of increased prey availability. In general, the benefit is quantified as a population-size increment of an upper-trophic-level species^1^. However, other benefits such as increased growth or foraging amounts can result from bottom-up effects. In particular, physiological benefits such as increased nutritional contributions to predators from prey have been relatively understudied in research on bottom-up effects despite that prey nutritional composition has the key role in predator growth and fitness^2^.

Many studies of bottom-up and top-down effects have focused on aquatic species because of the relative ease which with these effects can be detected in aquatic ecosystems^3^. However, the nutritional contributions of bottom-up effects have not been adequately studied even in these systems. Nonetheless, the nutritional compositions of several important fish species are well known^4^. For example, many fishes have high contents of long-chain omega-3 fatty acids including docosahexaenoic acid (DHA) and eicosapentaenoic acid (EPA)^4^. Because DHA is an important nutrient for growth, reproduction, and the functioning of sensory organs^5–7^, its availability could affect fish fitness. Fish accumulate DHA via the bioconversion of EPA contained within food resources or by consuming DHA-rich prey (e.g., other fish), and they accumulate EPA mainly by consuming invertebrates or insectivorous fish^8,9^.

Recent studies have used these traits of DHA and EPA in fishes to evaluate trophic levels in fish communities^10–12^. Although DHA and EPA contents can vary among individuals of the same species owing to differences in genetic background^13^, piscivorous fish tend to have high DHA content, and insectivorous/planktivorous fish tend to have high EPA content^10–12^. These differences are found both between species^10^ and between individuals of the same species that undergo ontogenetic shifts in diet niche^11,12^. Therefore, the DHA and EPA contents in fish could be a useful metric to test nutritional bottom-up effects.

**Figure 1:**
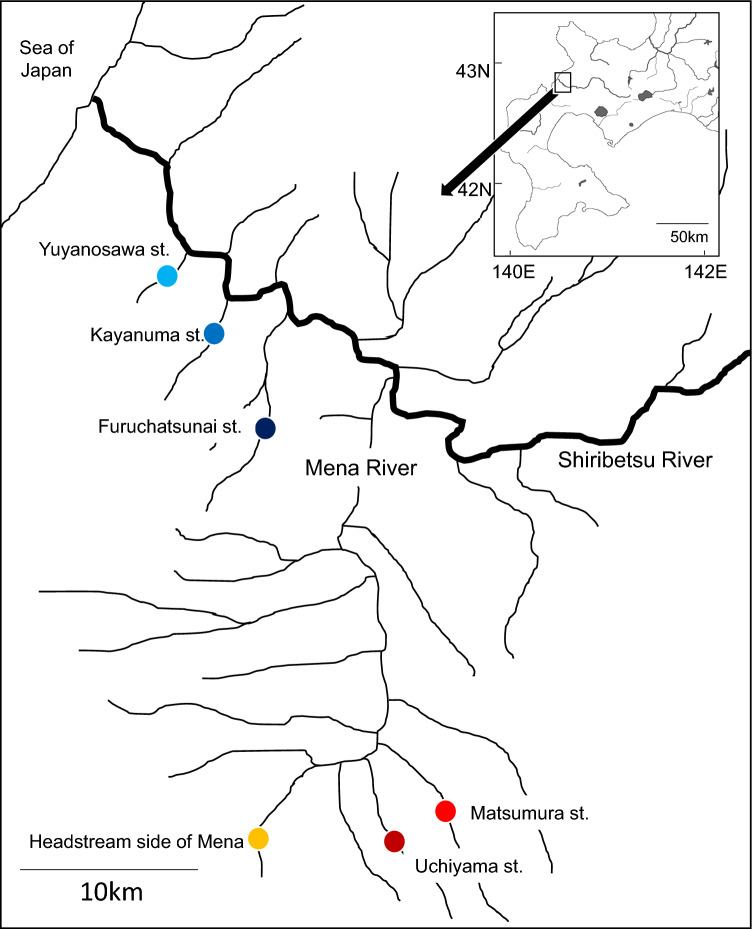
A map of sampling sites (circles) in the Shiribetsu River system. Sites shown with warm (yellow and reddish) colors are located on salmon-stocked streams; those shown with cool (bluish) colors are located on unstocked streams.

Hatchery programs, which stock large numbers of artificially reared fish into natural waters, provide a convenient system for evaluating bottom-up effects. For example, white-spotted charr *Salvelinus leucomaenis*, a salmonid fish, shifts its diet to stocked masu salmon *Oncorhynchus masou* fry in salmon-stocked streams; thus the growth of white-spotted charr in salmon-stocked streams is slightly higher than in unstocked streams^14^. In the present study, we test the hypothesis that fish stocking triggers a nutritional bottom-up effect by comparing DHA and EPA contents of white-spotted charr between salmon-stocked and unstocked streams (Fig. 1).

## Results

For all fatty acids (DHA/EPA/TFA), significant interaction terms were detected between study period and stream type (Table S2), suggesting that the differences between study periods (before and after stocking) varied between stocked and unstocked streams.

**Figure 2:**
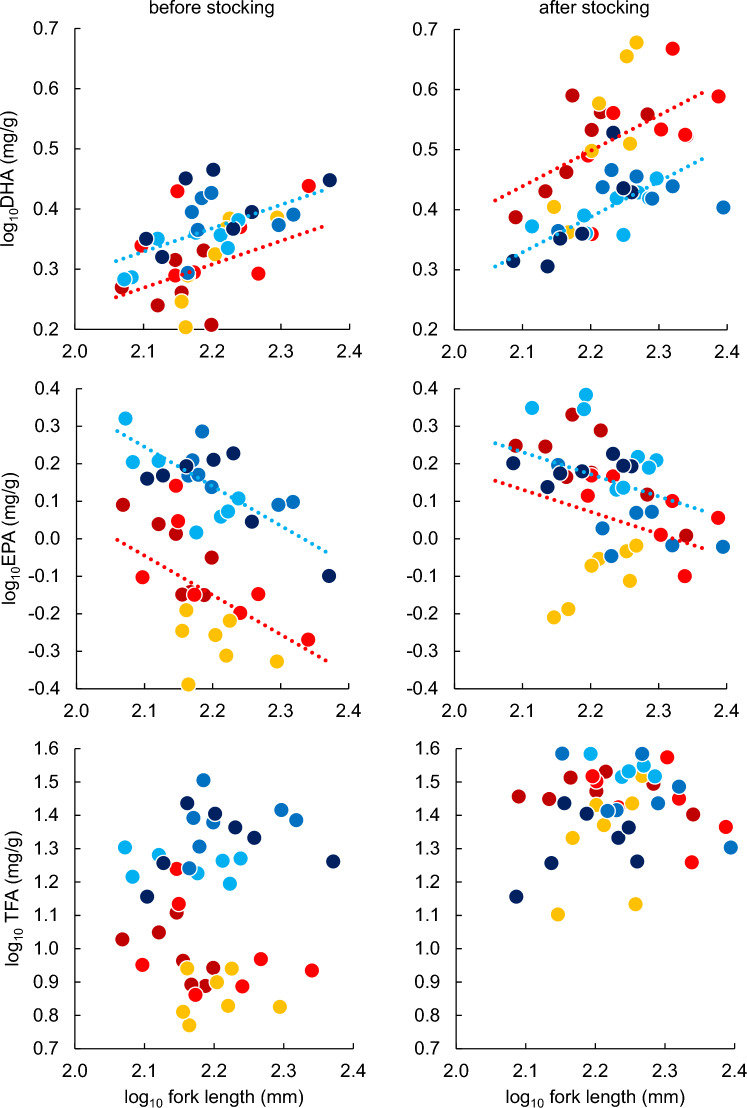
Relationships between fork length and (top) DHA, (middle) EPA, and (bottom) total fatty acid (TFA). Each plot indicates an experimental charr. The units of DHA, EPA, and TFA are mg-fatty-acid g-muscle^−1^. Left and right panels show relationships before and after salmon stocking, respectively. Colors indicate sampling sites, with warm colors corresponding to sites located in salmon-stocked streams, and cool colors to those in unstocked streams. Dotted lines are significant regression lines generated by using linear mixed models (red, salmon-stocked streams; blue, unstocked streams). Regression formulae are provided in Supplementary table S4.

DHA contents increased with increasing fork length (Table 1; Fig. 2). In stocked streams, DHA contents in charr were significantly higher after stocking than before stocking, whereas in unstocked streams, the difference of DHA contents were insignificant before and after stocking (Table 1; Fig. 2). As a result, the difference of DHA contents in charr were insignificant between stocked and unstocked streams before stocking, but DHA contents were significantly higher in stocked streams than in unstocked streams after stocking (Table S3; Fig. 2).

**Table 1:** The results of linear mixed models testing the effects of study period, fork length and their interaction terms on the DHA, EPA and TFA contents of white-spotted charr in stocked and unstocked streams.

	df_numerator_	df_denominator_	*F*	*p*
DHA				
Stocked streams				
Study period (SP)	1	40.00	66.63	< 0.001
Fork length (FL)	1	40.00	18.25	< 0.001
SP × FL	1	39.00	0.315	0.578
Unstocked streams				
Study period (SP)	1	38.57	2.112	0.154
Fork length (FL)	1	39.72	19.80	< 0.001
SP × FL	1	39.00	0.183	0.671
EPA				
Stocked streams				
Study period (SP)	1	38.09	69.02	< 0.001
Fork length (FL)	1	38.46	13.00	0.001
SP × FL	1	37.10	0.469	0.498
Unstocked streams				
Study period (SP)	1	38.37	2.583	0.116
Fork length (FL)	1	39.99	20.58	< 0.001
SP × FL	1	38.98	0.013	0.910
TFA				
Stocked streams				
Study period (SP)	1	39.01	216.5	< 0.001
Unstocked streams				
Study period (SP)	1	41.00	59.70	< 0.001

FL was not considered in TFA because a preliminary analysis revealed that FL did not correlate TFA (Table S2; Fig. 2).

In contrast to DHA, EPA contents decreased with increasing charr fork length (Table 1; Fig. 2). In stocked streams, EPA contents of charr after stocking were significantly higher than those before stocking, but the difference were insignificant between before and after stocking in unstocked streams (Table 1; Fig. 2). As a result, EPA contents were significantly lower in stocked streams than in unstocked streams before stocking, but the difference became insignificant after stocking (Table S3; Fig. 2).

In contrast to both DHA and EPA, TFA did not correlate with fork length (Table S2). TFA contents of charr significantly increased after stocking in both stocked and unstocked streams (Table 1; Fig. 2). However, the degree of increase was obviously larger in stocked streams than unstocked streams. As a result, TFA contents of charr in stocked streams were significantly lower than in unstocked streams before stocking, but the difference between stocked and unstocked streams was insignificant after stocking (Table S3; Fig. 2).

## Discussion

Our results show that the contents of long-chain omega-3 fatty acids (DHA, EPA, and TFA) increased after fry stocking in charr inhabiting stocked streams but not in charr inhabiting unstocked streams (the increase of TFA in unstocked streams after stocking was statistically significant, but the degree of increase was very small compared with stocked streams). Because we observed that charr in stocked streams prey heavily upon stocked salmon fry that are rich in DHA and EPA (Supplementary table S5), charr predation on salmon fry is the likely cause of the increase in fatty acid content in stocked streams. These results are consistent with our hypothesized nutritional bottom-up effect. The magnitude of the bottom-up effect in our study might have been overevaluation by the fact that the stocked salmon fry were fed artificial pellets containing fish meal during the rearing period.

One of the ways that fish acquire DHA is through synthesis from EPA^8, 15^. Thus, increased rates of DHA synthesis could conceivably reduce EPA content in fish tissues. However, the opposite relationships with fork length that we observed for DHA and EPA in this study are unexpected. The positive relationship between fork length and DHA may suggest that large charr are better able to synthesize DHA by consuming the larger amount of EPA. Because the relationship with fork length differed by each fatty acid (including DHA and EPA) (Supplementary table S1), there was no consistent relationship between fork length and TFA. Although our results suggest that charr can gain a large amount of fatty acids through predation on stocked salmon fry, further work is needed to determine how and whether these fatty acids contribute to charr growth and fitness.

The lower fatty acid content of charr in stocked streams before stocking as compared to unstocked streams was likely caused by interspecific competition with masu salmon fry stocked in prior years^14^. Although the charr sampled in this study might have been large enough to outcompete stocked masu salmon fry in interference competition, their foraging efficiency would nevertheless have been reduced in the presence of high densities of small, competitively inferior individuals. A similar effect was demonstrated that the foraging efficiency of wild masu salmon fry was reduced after stocking of chum salmon *Oncorhynchus keta* fry^16^. If this is indeed the case, the stocking of masu salmon fry could contribute to both an accumulation of fatty acids in charr through fry predation and a reduction of fatty acids through competition in stocked streams.

Freshwater ecosystems have been seriously altered by anthropogenic impacts, and previous studies have examined these alterations with a particular focus on community ecology^17^. Large-scale stocking of hatchery-reared fishes is known to alter trophic interactions^14,16^ and species compositions in stocked streams^18^. Physiological characteristics, including fatty acid composition, are closely tied to growth and fitness of wild fish, and previous studies suggest that these characteristics are also easily influenced by anthropogenic impacts (e.g., climate change^19^). The present study newly demonstrates a nutritional bottom-up effect that alters the physiology of fish inhabiting stocked streams. To obtain a comprehensive understanding of anthropogenic impacts on freshwater ecosystems, further studies should seek to link internal (physiological) changes among individuals to structural changes in community composition.

## Methods

### Study sites and salmon stocking program

The experimental fish were sampled in tributaries of the Shiribetsu River in Hokkaido, northern Japan in 2021 (Fig. 1). Artificially reared masu salmon fry were stocked into the Mena River in late May. During rearing in the hatchery, the fry were fed artificial pellets (Ambrose: Feed one Inc, Yokohama, Kanagawa, Japan) made from fish meal. White-spotted charr is native to the Shiribetsu River and is widely distributed in the river system. Thus, the species was a convenient study animal for evaluating the impact of stocked masu salmon fry.

Electrofishing (Model 12B; Smith-Root Inc, Vancouver, Washington, USA) was conducted to collect charr samples in salmon-stocked and unstocked streams before (20 May) and after (23 June) stocking. The stocked streams were the Uchiyama (number of fish stocked, 30,000; stocking date, 27 May; mean fork length, 47 mm) and Matsumura streams (30,000; 27 May; 47 mm) and the headstream side of the Mena River (131,000; 25 May; 46 mm) (Fig. 1). The unstocked streams were the Yuyanosawa, Kayanuma, and Furuchatsunai streams (Fig. 1). Masu salmon originating from natural spawning (i.e., wild fish) inhabited both the stocked and unstocked streams. In 2019, a previous study observed that charr predation on stocked masu salmon fry is time-limited, and charr do not prey upon the fry ca. 20 days after stocking^14^. We confirmed a similar pattern in this study, and no piscivory was observed in unstocked streams (Supplementary table S1).

### Fatty acid analysis

Only charr that were large enough to prey upon salmon fry demonstrated in the previous study^14^ were selected for fatty acid analysis (see Supplementary table S1 for fork lengths of experimental charr). The sampled fish were preserved on ice until transport to a laboratory at the Sapporo Field Station, Fisheries Research Institute. In the laboratory, they were kept in a freezer. Before fatty acid analysis, defrosted charr were measured for fork length to the nearest 1 mm.

A piece of tissue and skin (a cross section of muscle tissue on the side of the body) was sampled from the defrosted charr from the area between the gill cover and dorsal fin for lipid extraction. The sample tissues were weighed (approx. 1 g; nearest to 0.001 g) and quickly homogenized in chloroform/methanol (2:1, v/v). Total lipids were extracted by using the method described by Folch et al. (1957)^20^ and dissolved in a known volume of the fresh solvent. The total lipids were quantitatively transferred to a reaction tube and added to a known amount of docosanoic acid (22:0) as internal standard. Then, fatty acid methyl esters (FAMEs) were prepared by acid-catalyzed methylation using the procedure described by Ichihara & Fukubayashi (2010)^21^. The FAMEs were then analyzed by using gas chromatography (Agilent 8860GC, Santa Clara, California, USA) with an Omegawax 320 capillary column (length 30 m, internal diameter 0.32 mm, phase thickness 0.25 um) (Sigma-Aldrich Co. LCC, St. Louise, Missouri, USA) and a flame ionization detector. The initial column temperature was 190 °C, and the column was heated at 5 °C min^−1^ to 240 °C. Helium was used as the carrier gas. A known volume of FAME sample was injected using automatic sampler (Agilent 7693A). Chromatograms were integrated using Agilent OpenLAB data analysis software. FAMEs were identified by comparison of the relative times with known standards (Sigma-Aldrich Co. LCC). Quantification of fatty acids was calculated using the ratio of the FAME peak areas to the internal standard peak area, and the absolute amount of the internal standard (22:0) added. The quantified fatty acids were used to back calculated the mg of fatty acids per gram of muscle tissue of charr.

### Statistical analysis

To test whether charr fatty acid contents were affected by salmon stocking, linear mixed models (LMMs) with sampling site as a random effect were constructed as follows:

$$\begin{aligned} & {\text{DHA/EPA/TFA }}\;\left( {{\text{total}}\;{\text{ fatty}}\;{\text{ acid}}} \right)\; = \;{\text{study}}\;{\text{period}} + {\text{stream}}\;{\text{type}} + {\text{fork}}\;{\text{length }} + {\text{ study}}\;{\text{period}} \\ & \quad \quad \times {\text{ stream}}\;{\text{type}} + {\text{study}}\;{\text{ period }} \times {\text{ fork}}\;{\text{length}} + {\text{stream}}\;{\text{type}} \times {\text{fork}}\;{\text{length }} + {\text{ study}}\;{\text{period }} \times {\text{ stream}}\;{\text{type }} \times {\text{ fork}}\;{\text{length}} \\ \end{aligned}$$

For all fatty acids, significant interaction terms between study period × stream type were detected (Table S2). Then, LMMs with sampling site as a random effect as follows were conducted for stocked and unstocked streams, separately.

$${\text{DHA/EPA/TFA}}\left( {{\text{total}}\;{\text{fatty}}\;{\text{acid}}} \right) = {\text{study}}\;{\text{period }} + {\text{ fork}}\;{\text{ length }} + {\text{ study}}\;{\text{ period}} \times {\text{fork }}\;{\text{length}}$$

Fork length was not contained in the LMM for TFA because the preliminary analysis revealed that it did not correlate with TFA (Table S2). The LMMs replacing study period by stream type were also conducted, and its outputs were shown in Table S3.

Study period indicates the timing of sampling (before or after stocking), and stream type indicates whether stocking occurs (stocked or unstocked). Fork length was treated as a covariate because fatty acid contents sometimes covary with body size^11^. DHA/EPA/TFA and fork length were log_10_-transformed before analysis. If the highest-order interaction term was insignificant, the analysis was repeated without that interaction term.

Alpha level was set at 0.05. The sample sizes of experimental charr are shown in Supplementary table S1. All analyses were performed in SPSS version 24 (IBM Corp., Armonk, NY, USA).

### Ethical statement

All experimental protocols were approved by the ethics committee for the research animals of Fisheries Resources Institute, Japan Fisheries Research and Education Agency, and all methods were carried out in accordance with guidelines and regulations set out by the ethics committee. Also, all methods were carried out in accordance with the ARRIVE guidelines v2.0^22^. The authors obtained the legal permission from the governor of Hokkaido prefecture for fish samplings (number 158 of the permit for sampling organisms in the inland waters).

### Supplementary Information

Supplementary Tables.

## Data Availability

The raw data are provided in Supplementary table S1.
