# Supplementary material for: DHA and EPA levels in a piscivorous fish changed by preying upon stocked salmon fry
Source: Sci Rep. 2023 Sep 15;13:15278. doi: 10.1038/s41598-023-42530-2 (PMC10504290; doi:10.1038/s41598-023-42530-2)
Supplement: Supplementary file 1 — Supplementary Tables. [file 41598_2023_42530_MOESM1_ESM.pdf]

DHA and EPA levels in a piscivorous fish changed by preying upon stocked salmon fry

Koh Hasegawa, Yutaka Yano, Kentaro Honda, & Yuhei Ogura

Supplementary table S1. Fatty acid content (mg/g-muscle-tissue) and fork length (mm) of experimental charr. Pearson's r values show correlations between fork length and fatty acid contents for each stream type and study period (bold text indicates significant correlations at $p < 0.05$). "no. of masu salmon fry" indicates the number of masu salmon fry preyed upon by each charr. All preyed-upon masu salmon fry were regarded as stocked hatchery fish. The study period "just after stocking" refers to 31 May, which was 4 days after stocking in Uchiyama and Matsumura and 6 days after stocking for the headstream side of the Mena River. Fatty acid analyses were not conducted for fish sampled just after stocking.

charr ID	stream type	sampling site	study period	fork length	**TFA**	14:0	16:0	16:1 n7	18:0	18:1 n9	18:1 n7	18:2 n6	18:3 n3	18:4 n3	20:1 n9	20:1 n7	20:2 n6	20:3 n6	20:4 n6	20:4 n3	**20:5n (EPA)**	22:1 n11	22:1 n9	22:4 n6	22:5 n6	22:5 n3	**22:6n (DHA)**	no. of masu salmon fry
1	stocked	uchiyama	before stocking	154	7.74	0.11	1.38	0.31	0.44	0.72	0.18	0.32	0.36	0.12	0.00	0.02	0.03	0.04	0.39	0.07	0.71	0.00	0.00	0.04	0.09	0.26	2.14	0
2	stocked	uchiyama	before stocking	147	7.81	0.16	1.46	0.45	0.43	0.94	0.18	0.46	0.51	0.13	0.00	0.02	0.03	0.05	0.32	0.08	0.72	0.00	0.00	0.03	0.08	0.24	1.51	0
3	stocked	uchiyama	before stocking	158	8.76	0.15	1.68	0.52	0.45	0.95	0.26	0.52	0.49	0.16	0.00	0.02	0.05	0.07	0.44	0.10	0.89	0.00	0.00	0.04	0.11	0.26	1.61	0
4	stocked	uchiyama	before stocking	143	9.20	0.12	1.64	0.43	0.52	1.30	0.22	0.75	0.49	0.08	0.02	0.03	0.05	0.07	0.50	0.07	0.71	0.00	0.00	0.05	0.12	0.20	1.82	0
5	stocked	uchiyama	before stocking	140	12.84	0.28	2.38	0.93	0.62	1.95	0.43	0.98	0.72	0.26	0.02	0.04	0.05	0.08	0.41	0.13	1.03	0.00	0.00	0.04	0.08	0.32	2.07	0
6	stocked	uchiyama	before stocking	132	11.19	0.19	2.04	0.65	0.61	1.69	0.42	0.78	0.75	0.14	0.05	0.02	0.05	0.07	0.45	0.11	1.09	0.00	0.00	0.00	0.08	0.28	1.74	0
7	stocked	uchiyama	before stocking	117	10.67	0.27	2.06	0.76	0.51	1.05	0.33	0.57	0.75	0.26	0.00	0.02	0.03	0.06	0.39	0.14	1.23	0.00	0.00	0.00	0.06	0.31	1.86	0
mean				142	9.74	0.18	1.81	0.58	0.51	1.23	0.29	0.63	0.58	0.16	0.01	0.03	0.04	0.06	0.42	0.10	0.91	0.00	0.00	0.03	0.09	0.26	1.82	0
SD				14	1.90	0.07	0.36	0.21	0.08	0.44	0.11	0.23	0.16	0.07	0.02	0.01	0.01	0.01	0.06	0.03	0.21	0.00	0.00	0.02	0.02	0.04	0.23	0
8	stocked	matsumura	before stocking	219	8.60	0.05	1.60	0.29	0.44	1.00	0.18	0.51	0.38	0.03	0.00	0.03	0.04	0.04	0.30	0.05	0.54	0.00	0.00	0.04	0.12	0.22	2.74	0
9	stocked	matsumura	before stocking	185	9.31	0.09	1.68	0.41	0.48	1.29	0.27	0.71	0.57	0.08	0.02	0.03	0.06	0.06	0.45	0.07	0.71	0.00	0.00	0.05	0.10	0.21	1.96	0
10	stocked	matsumura	before stocking	174	7.71	0.07	1.43	0.25	0.39	0.83	0.18	0.35	0.29	0.05	0.00	0.02	0.04	0.06	0.38	0.05	0.63	0.00	0.00	0.04	0.10	0.20	2.34	0
11	stocked	matsumura	before stocking	149	7.27	0.07	1.35	0.27	0.38	0.66	0.18	0.33	0.28	0.05	0.00	0.02	0.03	0.07	0.45	0.06	0.71	0.00	0.00	0.06	0.11	0.22	1.97	0
12	stocked	matsumura	before stocking	140	17.35	0.44	2.97	1.35	0.83	2.93	0.61	1.34	1.46	0.31	0.06	0.08	0.08	0.12	0.57	0.19	1.38	0.02	0.00	0.07	0.19	0.41	1.95	0
13	stocked	matsumura	before stocking	141	13.63	0.36	2.56	1.07	0.66	1.55	0.43	0.85	0.77	0.26	0.04	0.02	0.07	0.08	0.54	0.14	1.11	0.00	0.00	0.00	0.13	0.28	2.69	0
14	stocked	matsumura	before stocking	125	8.94	0.16	1.61	0.54	0.46	1.01	0.30	0.45	0.45	0.11	0.04	0.03	0.06	0.05	0.31	0.07	0.79	0.00	0.00	0.03	0.06	0.23	2.18	0
mean				162	10.40	0.18	1.89	0.60	0.52	1.33	0.31	0.65	0.60	0.13	0.02	0.03	0.05	0.07	0.43	0.09	0.84	0.00	0.00	0.04	0.11	0.25	2.26	0
SD				33	3.70	0.16	0.62	0.44	0.17	0.77	0.16	0.36	0.42	0.11	0.02	0.02	0.02	0.02	0.11	0.05	0.30	0.01	0.00	0.02	0.04	0.07	0.34	0
15	stocked	kamimena	before stocking	197	6.70	0.05	1.22	0.14	0.39	0.67	0.12	0.21	0.13	0.03	0.00	0.02	0.03	0.04	0.37	0.04	0.47	0.00	0.00	0.08	0.08	0.17	2.43	0
16	stocked	kamimena	before stocking	168	8.72	0.11	1.50	0.33	0.55	1.36	0.19	0.37	0.28	0.09	0.00	0.04	0.03	0.05	0.40	0.06	0.60	0.00	0.00	0.04	0.09	0.20	2.42	0
17	stocked	kamimena	before stocking	160	7.94	0.12	1.32	0.32	0.49	1.10	0.20	0.36	0.23	0.18	0.00	0.03	0.03	0.05	0.36	0.10	0.55	0.00	0.00	0.10	0.09	0.19	2.11	0
18	stocked	kamimena	before stocking	166	6.74	0.06	1.18	0.19	0.41	0.75	0.12	0.23	0.12	0.06	0.00	0.02	0.02	0.04	0.31	0.05	0.49	0.00	0.00	0.05	0.10	0.20	2.33	0
19	stocked	kamimena	before stocking	146	5.89	0.07	1.04	0.16	0.36	0.55	0.11	0.22	0.16	0.07	0.00	0.00	0.03	0.04	0.29	0.05	0.41	0.00	0.00	0.09	0.10	0.21	1.95	0
20	stocked	kamimena	before stocking	143	6.47	0.08	1.18	0.22	0.38	0.72	0.12	0.31	0.24	0.02	0.00	0.02	0.04	0.06	0.43	0.07	0.57	0.00	0.00	0.00	0.10	0.16	1.76	0
21	stocked	kamimena	before stocking	145	8.73	0.08	1.51	0.39	0.45	1.18	0.28	0.81	0.49	0.12	0.00	0.04	0.05	0.10	0.49	0.10	0.64	0.00	0.00	0.05	0.11	0.26	1.60	0
mean				161	7.31	0.08	1.28	0.25	0.43	0.90	0.16	0.36	0.24	0.08	0.00	0.02	0.03	0.05	0.38	0.07	0.53	0.00	0.00	0.06	0.10	0.20	2.09	0
SD				19	1.14	0.03	0.18	0.10	0.07	0.31	0.06	0.21	0.13	0.05	0.00	0.01	0.01	0.02	0.07	0.02	0.08	0.00	0.00	0.03	0.01	0.03	0.33	0
mean (total)				155	9.15	0.15	1.66	0.48	0.49	1.15	0.25	0.54	0.47	0.12	0.01	0.03	0.04	0.06	0.41	0.09	0.76	0.00	0.00	0.04	0.10	0.24	2.06	0
SD (total)				24	2.73	0.11	0.49	0.32	0.11	0.55	0.13	0.29	0.31	0.08	0.02	0.01	0.02	0.02	0.08	0.04	0.27	0.00	0.00	0.03	0.03	0.06	0.34	0
				pearson's r	-0.37	**-0.66**	-0.37	**-0.55**	-0.34	-0.26	**-0.47**	-0.35	**-0.47**	**-0.55**	-0.63	-0.02	-0.29	**-0.50**	-0.27	**-0.64**	**-0.60**			0.06	0.26	**-0.50**	**0.50**	

Supplementary table S1 (continued). Fatty acid content (mg/g-muscle-tissue) and fork length (mm) of experimental charr. Pearson's r values show correlations between fork length and fatty acid contents for each stream type and study period (bold text indicates significant correlations at $p < 0.05$). "no. of masu salmon fry" indicates the number of masu salmon fry preyed upon by each charr. All preyed-upon masu salmon fry were regarded as stocked hatchery fish. The study period "just after stocking" refers to 31 May, which was 4 days after stocking in Uchiyama and Matsumura and 6 days after stocking for the headstream side of the Mena River. Fatty acid analyses were not conducted for fish sampled just after stocking.

charr ID	stream type	sampling site	study period	fork length	**TFA**	14:0	16:0	16:1 n7	18:0	18:1 n9	18:1 n7	18:2 n6	18:3 n3	18:4 n3	20:1 n9	20:1 n7	20:2 n6	20:3 n6	20:4 n6	20:4 n3	**20:5n (EPA)**	22:1 n11	22:1 n9	22:4 n6	22:5 n6	22:5 n3	**22:6n (DHA)**	no. of masu salmon fry
22	unstocked	yuyanosawa	before stocking	173	18.64	0.69	3.12	1.55	0.89	3.17	0.73	1.55	1.30	0.23	0.06	0.10	0.13	0.11	0.53	0.13	1.28	0.02	0.02	0.07	0.11	0.44	2.41	0
23	unstocked	yuyanosawa	before stocking	163	18.36	0.46	3.12	1.36	0.82	3.46	0.65	1.88	1.18	0.19	0.06	0.12	0.16	0.15	0.56	0.14	1.15	0.02	0.02	0.07	0.15	0.38	2.27	0
24	unstocked	yuyanosawa	before stocking	167	15.67	0.24	2.68	0.96	0.75	2.55	0.60	1.56	0.92	0.15	0.06	0.10	0.17	0.13	0.61	0.12	1.18	0.02	0.02	0.08	0.16	0.43	2.16	0
25	unstocked	yuyanosawa	before stocking	150	16.83	0.23	3.02	1.15	0.85	3.03	0.64	1.52	1.08	0.18	0.08	0.09	0.13	0.15	0.61	0.12	1.04	0.04	0.02	0.08	0.17	0.34	2.29	0
26	unstocked	yuyanosawa	before stocking	132	19.11	0.49	3.57	1.59	0.88	3.02	0.86	1.57	1.37	0.28	0.04	0.07	0.12	0.11	0.52	0.16	1.61	0.00	0.00	0.06	0.13	0.40	2.24	0
27	unstocked	yuyanosawa	before stocking	121	16.43	0.36	3.25	1.34	0.72	2.19	0.80	1.24	1.36	0.27	0.03	0.06	0.09	0.10	0.47	0.15	1.60	0.00	0.00	0.06	0.09	0.33	1.93	0
28	unstocked	yuyanosawa	before stocking	118	20.13	0.47	4.04	2.10	0.89	2.44	1.24	1.25	1.69	0.42	0.04	0.06	0.09	0.11	0.53	0.19	2.09	0.02	0.00	0.04	0.10	0.42	1.92	0
mean				146	17.88	0.42	3.26	1.44	0.83	2.84	0.79	1.51	1.27	0.24	0.05	0.09	0.13	0.12	0.54	0.15	1.42	0.02	0.01	0.06	0.13	0.39	2.18	0
SD				23	1.61	0.16	0.43	0.36	0.07	0.45	0.22	0.22	0.24	0.09	0.02	0.02	0.03	0.02	0.05	0.02	0.37	0.01	0.01	0.01	0.03	0.04	0.19	0
29	unstocked	kayanuma	before stocking	208	24.30	0.29	3.64	1.44	1.18	5.41	0.84	3.15	1.64	0.12	0.04	0.23	0.34	0.17	0.86	0.15	1.25	0.02	0.04	0.20	0.34	0.50	2.46	0
30	unstocked	kayanuma	before stocking	198	26.05	0.36	4.25	1.91	1.14	6.05	0.97	3.00	1.82	0.18	0.04	0.23	0.29	0.18	0.88	0.16	1.23	0.02	0.04	0.16	0.29	0.50	2.36	0
31	unstocked	kayanuma	before stocking	153	32.01	0.71	5.24	2.51	1.51	6.65	1.30	3.87	2.27	0.22	0.05	0.26	0.39	0.21	0.94	0.19	1.93	0.02	0.05	0.15	0.30	0.60	2.62	0
32	unstocked	kayanuma	before stocking	148	24.68	0.35	4.52	1.96	1.14	4.57	1.09	2.25	2.19	0.21	0.02	0.14	0.19	0.13	0.77	0.17	1.62	0.00	0.03	0.12	0.25	0.49	2.48	0
33	unstocked	kayanuma	before stocking	158	23.93	0.53	4.11	1.64	1.15	4.55	0.91	2.69	1.45	0.19	0.05	0.20	0.31	0.18	0.82	0.14	1.37	0.02	0.03	0.12	0.39	0.42	2.67	0
34	unstocked	kayanuma	before stocking	146	17.43	0.48	3.29	1.53	0.89	2.49	0.85	1.40	1.22	0.12	0.05	0.08	0.14	0.10	0.62	0.11	1.47	0.02	0.00	0.06	0.11	0.41	1.97	0
35	unstocked	kayanuma	before stocking	151	20.25	0.26	3.39	1.22	0.98	3.75	0.76	2.26	1.43	0.16	0.04	0.15	0.24	0.14	0.75	0.14	1.48	0.03	0.00	0.12	0.18	0.43	2.32	0
mean				166	24.09	0.43	4.06	1.75	1.14	4.78	0.96	2.66	1.72	0.17	0.04	0.19	0.27	0.16	0.81	0.15	1.48	0.02	0.03	0.13	0.26	0.48	2.41	0
SD				26	4.59	0.16	0.69	0.43	0.19	1.41	0.18	0.79	0.40	0.04	0.01	0.06	0.09	0.04	0.10	0.02	0.24	0.01	0.02	0.04	0.09	0.07	0.23	0
36	unstocked	furuchatsunai	before stocking	235	18.26	0.22	2.64	1.03	0.89	4.03	0.57	1.76	0.74	0.12	0.06	0.16	0.14	0.15	0.64	0.10	0.80	0.02	0.03	0.20	0.62	0.57	2.80	0
37	unstocked	furuchatsunai	before stocking	181	21.50	0.92	3.39	1.67	1.01	3.80	0.90	2.12	1.04	0.20	0.08	0.13	0.17	0.15	0.68	0.12	1.11	0.03	0.03	0.24	0.45	0.78	2.48	0
38	unstocked	furuchatsunai	before stocking	170	23.10	0.43	3.97	1.90	1.19	3.93	1.16	1.87	1.63	0.30	0.11	0.13	0.20	0.19	0.85	0.22	1.69	0.04	0.02	0.14	0.56	0.27	2.33	0
39	unstocked	furuchatsunai	before stocking	159	25.40	0.42	4.32	2.39	1.30	4.81	1.21	1.95	1.63	0.34	0.10	0.15	0.15	0.15	0.70	0.20	1.62	0.03	0.03	0.11	0.64	0.24	2.92	0
40	unstocked	furuchatsunai	before stocking	145	27.31	0.77	4.53	2.21	1.39	5.41	1.35	2.48	1.55	0.29	0.12	0.18	0.24	0.20	0.83	0.20	1.56	0.04	0.03	0.21	0.59	0.31	2.82	0
41	unstocked	furuchatsunai	before stocking	134	18.07	0.73	3.02	1.45	0.88	3.01	0.93	1.47	0.92	0.20	0.07	0.10	0.14	0.14	0.65	0.13	1.47	0.02	0.00	0.07	0.13	0.43	2.09	0
42	unstocked	furuchatsunai	before stocking	127	14.32	0.55	2.41	1.05	0.68	1.96	0.67	0.91	0.76	0.18	0.04	0.05	0.08	0.10	0.51	0.13	1.45	0.00	0.00	0.08	0.11	0.37	2.24	0
mean				164	21.14	0.58	3.47	1.67	1.05	3.85	0.97	1.80	1.18	0.23	0.08	0.13	0.16	0.15	0.69	0.16	1.39	0.03	0.02	0.15	0.44	0.42	2.53	0
SD				37	4.55	0.24	0.83	0.53	0.25	1.13	0.29	0.50	0.41	0.08	0.03	0.04	0.05	0.03	0.12	0.05	0.32	0.01	0.01	0.07	0.23	0.19	0.32	0
mean (total)				159	21.04	0.47	3.60	1.62	1.01	3.82	0.91	1.99	1.39	0.22	0.06	0.13	0.19	0.14	0.68	0.15	1.43	0.02	0.02	0.12	0.28	0.43	2.37	0
SD (total)				29	4.48	0.20	0.73	0.44	0.22	1.31	0.24	0.72	0.42	0.08	0.03	0.06	0.09	0.03	0.14	0.03	0.30	0.01	0.02	0.06	0.19	0.12	0.28	0
				pearson's r	0.27	-0.31	-0.05	-0.14	0.30	**0.56**	-0.27	**0.52**	-0.09	**-0.55**	0.19	**0.67**	**0.52**	**0.54**	**0.46**	-0.29	**-0.70**	-0.09	0.16	**0.69**	**0.62**	0.40	**0.56**	

Supplementary table S1 (continued). Fatty acid content (mg/g-muscle-tissue) and fork length (mm) of experimental charr. Pearson's r values show correlations between fork length and fatty acid contents for each stream type and study period (bold text indicates significant correlations at $p < 0.05$). "no. of masu salmon fry" indicates the number of masu salmon fry preyed upon by each charr. All preyed-upon masu salmon fry were regarded as stocked hatchery fish. The study period "just after stocking" refers to 31 May, which was 4 days after stocking in Uchiyama and Matsumura and 6 days after stocking for the headstream side of the Mena River. Fatty acid analyses were not conducted for fish sampled just after stocking.

charr ID	stream type	sampling site	study period	fork length	**TFA**	14:0	16:0	16:1 n7	18:0	18:1 n9	18:1 n7	18:2 n6	18:3 n3	18:4 n3	20:1 n9	20:1 n7	20:2 n6	20:3 n6	20:4 n6	20:4 n3	**20:5n (EPA)**	22:1 n11	22:1 n9	22:4 n6	22:5 n6	22:5 n3	**22:6n (DHA)**	no. of masu salmon fry
43	stocked	uchiyama	after stocking	219	25.27	0.45	4.89	1.77	1.28	5.05	0.88	1.99	2.28	0.31	0.05	0.22	0.17	0.13	0.54	0.19	1.02	0.00	0.00	0.00	0.18	0.53	3.33	0
44	stocked	uchiyama	after stocking	159	29.65	0.61	5.51	2.16	1.39	5.26	1.04	2.36	3.43	0.52	0.06	0.22	0.16	0.21	0.55	0.41	1.50	0.00	0.00	0.00	0.21	0.63	3.41	0
45	stocked	uchiyama	after stocking	192	31.25	0.62	6.07	2.13	1.43	6.30	0.98	2.45	3.52	0.42	0.09	0.25	0.17	0.17	0.59	0.24	1.31	0.00	0.00	0.15	0.40	0.33	3.62	0
46	stocked	uchiyama	after stocking	164	33.97	0.97	6.79	3.03	1.56	5.96	1.35	2.46	3.06	0.62	0.25	0.17	0.17	0.21	0.67	0.30	1.94	0.00	0.00	0.00	0.22	0.58	3.65	0
47	stocked	uchiyama	after stocking	149	43.51	0.94	8.40	3.33	1.93	8.18	1.43	3.24	5.98	1.03	0.07	0.28	0.19	0.18	0.58	0.57	2.14	0.00	0.00	0.00	0.26	0.88	3.89	0
48	stocked	uchiyama	after stocking	146	32.59	0.69	6.39	2.22	1.53	6.30	1.00	2.78	4.89	0.66	0.22	0.04	0.17	0.15	0.46	0.30	1.46	0.03	0.00	0.00	0.02	0.37	2.90	0
49	stocked	uchiyama	after stocking	123	28.62	0.77	5.36	2.44	1.28	4.60	1.09	2.11	4.23	0.87	0.15	0.03	0.11	0.15	0.36	0.34	1.77	0.03	0.00	0.00	0.05	0.44	2.44	0
50	stocked	uchiyama	after stocking	136	28.11	0.82	5.53	2.27	1.25	4.66	1.06	1.82	3.69	0.73	0.04	0.18	0.10	0.14	0.42	0.35	1.76	0.03	0.00	0.08	0.07	0.41	2.70	0
mean				161	31.62	0.73	6.12	2.42	1.46	5.79	1.10	2.40	3.89	0.65	0.12	0.17	0.16	0.17	0.52	0.34	1.61	0.01	0.00	0.03	0.18	0.52	3.24	0
SD				31	5.52	0.18	1.10	0.51	0.22	1.18	0.19	0.46	1.14	0.24	0.08	0.09	0.03	0.03	0.10	0.12	0.36	0.02	0.00	0.06	0.13	0.18	0.51	0
51	stocked	matsumura	after stocking	244	23.18	0.47	4.35	1.58	1.14	4.37	0.83	1.76	1.64	0.24	0.04	0.17	0.14	0.14	0.61	0.21	1.14	0.00	0.00	0.00	0.12	0.37	3.88	0
52	stocked	matsumura	after stocking	209	28.20	0.56	5.48	2.26	1.28	5.78	1.08	2.00	1.52	0.25	0.10	0.26	0.16	0.14	0.51	0.21	1.26	0.00	0.00	0.00	0.16	0.53	4.66	0
53	stocked	matsumura	after stocking	218	18.16	0.29	3.41	0.86	0.90	3.15	0.51	1.50	1.73	0.16	0.05	0.13	0.10	0.11	0.48	0.14	0.80	0.00	0.00	0.00	0.12	0.38	3.35	0
54	stocked	matsumura	after stocking	201	37.49	0.44	7.44	2.01	1.45	8.04	0.69	3.15	6.98	0.57	0.07	0.28	0.25	0.17	0.51	0.30	1.02	0.00	0.00	0.00	0.20	0.51	3.42	0
55	stocked	matsumura	after stocking	171	26.56	0.50	5.24	2.07	1.18	4.88	0.92	1.90	2.27	0.35	0.06	0.19	0.16	0.16	0.59	0.23	1.47	0.00	0.00	0.00	0.20	0.55	3.64	0
56	stocked	matsumura	after stocking	159	31.77	0.56	5.81	2.26	1.35	6.33	1.05	2.88	5.38	0.57	0.25	0.06	0.19	0.14	0.46	0.30	1.47	0.04	0.00	0.00	0.02	0.36	2.29	0
57	stocked	matsumura	after stocking	157	32.91	0.56	6.84	2.32	1.46	7.34	1.06	2.82	3.96	0.44	0.25	0.04	0.18	0.15	0.47	0.24	1.30	0.04	0.00	0.00	0.02	0.34	3.09	0
mean				194	28.32	0.48	5.51	1.91	1.25	5.70	0.88	2.29	3.35	0.37	0.12	0.16	0.17	0.15	0.52	0.23	1.21	0.01	0.00	0.00	0.12	0.43	3.47	0
SD				33	6.45	0.10	1.38	0.53	0.20	1.71	0.22	0.65	2.15	0.16	0.09	0.09	0.05	0.02	0.06	0.06	0.24	0.02	0.00	0.00	0.07	0.09	0.73	0
58	stocked	kamimena	after stocking	179	27.28	0.48	5.53	1.55	1.43	5.87	0.71	2.00	2.17	0.32	0.11	0.26	0.16	0.13	0.46	0.24	0.93	0.00	0.00	0.00	0.00	0.40	4.52	0
59	stocked	kamimena	after stocking	185	32.97	0.58	6.91	1.87	1.56	7.64	0.77	2.79	2.46	0.29	0.06	0.36	0.25	0.24	0.59	0.23	0.96	0.00	0.00	0.00	0.21	0.43	4.77	0
60	stocked	kamimena	after stocking	163	23.49	0.51	4.38	1.36	1.30	5.17	0.62	1.88	1.10	0.41	0.06	0.27	0.18	0.20	0.56	0.20	0.88	0.00	0.00	0.00	0.19	0.45	3.78	0
61	stocked	kamimena	after stocking	181	13.60	0.17	2.33	0.60	0.74	2.43	0.36	0.85	0.47	0.10	0.05	0.13	0.15	0.11	0.38	0.10	0.77	0.00	0.00	0.00	0.13	0.49	3.24	0
62	stocked	kamimena	after stocking	159	26.98	0.37	5.63	1.22	1.25	6.01	0.55	2.57	2.94	0.33	0.05	0.24	0.21	0.21	0.50	0.23	0.85	0.00	0.00	0.13	0.17	0.39	3.15	0
63	stocked	kamimena	after stocking	147	21.49	0.20	4.09	0.80	1.02	4.22	0.41	2.27	3.67	0.37	0.14	0.03	0.14	0.13	0.51	0.17	0.65	0.03	0.00	0.00	0.13	0.20	2.30	0
64	stocked	kamimena	after stocking	140	12.67	0.24	2.35	0.46	0.67	1.97	0.25	0.90	1.32	0.38	0.06	0.00	0.05	0.08	0.32	0.17	0.62	0.02	0.00	0.00	0.21	0.08	2.54	0
mean				165	22.64	0.36	4.46	1.12	1.14	4.76	0.52	1.89	2.02	0.31	0.08	0.19	0.16	0.16	0.47	0.19	0.81	0.01	0.00	0.02	0.15	0.35	3.47	0
SD				18	7.42	0.16	1.71	0.52	0.34	2.03	0.19	0.76	1.12	0.10	0.04	0.14	0.06	0.06	0.10	0.05	0.13	0.01	0.00	0.05	0.07	0.15	0.94	0
mean (total)				173	27.71	0.54	5.40	1.84	1.29	5.43	0.85	2.20	3.12	0.45	0.10	0.17	0.16	0.16	0.50	0.26	1.23	0.01	0.00	0.02	0.15	0.44	3.39	0
SD (total)				31	7.23	0.22	1.51	0.74	0.28	1.64	0.31	0.64	1.66	0.23	0.07	0.10	0.05	0.04	0.09	0.10	0.43	0.02	0.00	0.04	0.10	0.16	0.71	0
				pearson's r	-0.08	-0.21	-0.07	-0.07	-0.06	0.04	-0.04	-0.08	-0.34	**-0.63**	-0.38	**0.51**	0.27	-0.01	**0.48**	-0.42	-0.27	**0.82**		0.95	0.35	0.23	**0.60**	

Supplementary table S1 (continued). Fatty acid content (mg/g-muscle-tissue) and fork length (mm) of experimental charr. Pearson's r values show correlations between fork length and fatty acid contents for each stream type and study period (bold text indicates significant correlations at $p < 0.05$). "no. of masu salmon fry" indicates the number of masu salmon fry preyed upon by each charr. All preyed-upon masu salmon fry were regarded as stocked hatchery fish. The study period "just after stocking" refers to 31 May, which was 4 days after stocking in Uchiyama and Matsumura and 6 days after stocking for the headstream side of the Mena River. Fatty acid analyses were not conducted for fish sampled just after stocking.

charr ID	stream type	sampling site	study period	fork length	**TFA**	14:0	16:0	16:1 n7	18:0	18:1 n9	18:1 n7	18:2 n6	18:3 n3	18:4 n3	20:1 n9	20:1 n7	20:2 n6	20:3 n6	20:4 n6	20:4 n3	**20:5n (EPA)**	22:1 n11	22:1 n9	22:4 n6	22:5 n6	22:5 n3	**22:6n (DHA)**	no. of masu salmon fry
65	unstocked	yuyanosawa	after stocking	198	45.46	0.85	8.87	3.37	2.11	10.21	1.40	4.07	6.46	0.85	0.05	0.31	0.30	0.24	0.71	0.38	1.62	0.00	0.00	0.00	0.33	0.48	2.83	0
66	unstocked	yuyanosawa	after stocking	186	35.41	0.51	6.33	2.79	1.47	7.70	1.23	3.53	4.27	0.50	0.05	0.22	0.29	0.21	0.68	0.30	1.65	0.00	0.00	0.00	0.34	0.65	2.68	0
67	unstocked	yuyanosawa	after stocking	193	32.89	0.59	5.92	2.69	1.62	7.20	1.18	3.43	3.34	0.40	0.04	0.23	0.24	0.19	0.66	0.23	1.55	0.00	0.00	0.00	0.33	0.44	2.62	0
68	unstocked	yuyanosawa	after stocking	173	32.76	0.44	6.58	2.08	1.51	6.33	1.04	2.74	5.17	0.61	0.04	0.21	0.22	0.19	0.67	0.30	1.35	0.00	0.00	0.00	0.24	0.39	2.63	0
69	unstocked	yuyanosawa	after stocking	177	34.06	0.35	6.45	1.85	1.34	7.48	0.89	3.78	5.55	0.49	0.04	0.20	0.25	0.18	0.60	0.28	1.37	0.00	0.00	0.00	0.25	0.42	2.28	0
70	unstocked	yuyanosawa	after stocking	156	38.40	0.69	7.58	3.62	1.55	7.15	1.80	3.15	5.32	0.74	0.21	0.05	0.18	0.17	0.56	0.33	2.42	0.03	0.00	0.00	0.54	0.02	2.29	0
71	unstocked	yuyanosawa	after stocking	155	47.54	0.70	9.45	3.86	1.98	10.48	1.67	3.92	7.44	0.93	0.33	0.05	0.22	0.21	0.60	0.39	2.22	0.04	0.00	0.00	0.57	0.03	2.46	0
72	unstocked	yuyanosawa	after stocking	130	48.01	0.65	9.55	4.02	1.81	10.70	1.65	3.94	7.62	1.05	0.06	0.28	0.21	0.20	0.47	0.44	2.23	0.05	0.00	0.09	0.07	0.58	2.36	0
mean				171	39.32	0.60	7.59	3.04	1.67	8.41	1.36	3.57	5.65	0.70	0.10	0.19	0.24	0.20	0.62	0.33	1.80	0.02	0.00	0.01	0.33	0.38	2.52	0
SD				23	6.64	0.16	1.49	0.81	0.27	1.75	0.33	0.45	1.48	0.23	0.11	0.10	0.04	0.02	0.08	0.07	0.42	0.02	0.00	0.03	0.16	0.23	0.20	0
73	unstocked	kayanuma	after stocking	248	20.12	0.23	3.49	1.24	0.88	4.13	0.51	1.97	1.59	0.16	0.03	0.15	0.23	0.16	0.73	0.16	0.95	0.00	0.00	0.16	0.14	0.70	2.53	0
74	unstocked	kayanuma	after stocking	209	30.63	0.25	6.06	1.43	1.31	6.84	0.53	2.96	4.68	0.59	0.04	0.21	0.20	0.18	0.56	0.26	0.96	0.00	0.00	0.17	0.13	0.52	2.75	0
75	unstocked	kayanuma	after stocking	185	38.39	0.45	7.39	2.15	1.76	8.55	0.80	3.49	6.62	0.49	0.04	0.31	0.29	0.23	0.59	0.35	1.17	0.00	0.00	0.16	0.12	0.57	2.85	0
76	unstocked	kayanuma	after stocking	195	27.30	0.39	5.00	1.59	1.31	5.52	0.81	2.75	3.16	0.35	0.05	0.19	0.21	0.22	0.69	0.20	1.18	0.00	0.00	0.20	0.18	0.69	2.62	0
77	unstocked	kayanuma	after stocking	170	26.03	0.29	4.85	1.29	1.15	5.88	0.52	2.55	3.11	0.46	0.06	0.19	0.22	0.17	0.51	0.22	0.90	0.00	0.00	0.00	0.15	0.61	2.92	0
78	unstocked	kayanuma	after stocking	165	25.90	0.33	4.36	1.63	1.32	6.08	0.79	2.67	2.63	0.23	0.22	0.04	0.24	0.17	0.66	0.17	1.07	0.04	0.00	0.00	0.49	0.02	2.74	0
79	unstocked	kayanuma	after stocking	142	38.48	0.52	7.55	2.48	1.61	7.78	1.00	3.64	7.07	0.76	0.21	0.03	0.25	0.19	0.61	0.37	1.57	0.04	0.00	0.00	0.46	0.03	2.32	0
mean				188	29.55	0.35	5.53	1.69	1.33	6.40	0.71	2.86	4.12	0.43	0.09	0.16	0.23	0.19	0.62	0.25	1.11	0.01	0.00	0.10	0.24	0.45	2.68	0
SD				34	6.82	0.11	1.53	0.46	0.29	1.47	0.19	0.57	2.08	0.21	0.08	0.10	0.03	0.03	0.08	0.09	0.23	0.02	0.00	0.09	0.16	0.30	0.21	0
80	unstocked	furuchatsunai	after stocking	182	45.33	0.65	8.81	4.09	2.10	10.04	1.65	4.74	5.08	0.77	0.12	0.30	0.36	0.32	0.95	0.35	1.56	0.00	0.00	0.12	0.18	0.46	2.68	0
81	unstocked	furuchatsunai	after stocking	171	45.20	0.59	8.25	3.01	2.04	10.19	1.41	4.43	5.90	0.81	0.06	0.33	0.36	0.30	0.91	0.41	1.68	0.00	0.00	0.24	0.18	0.72	3.37	0
82	unstocked	furuchatsunai	after stocking	177	40.05	0.64	7.19	3.02	1.86	8.52	1.49	4.30	4.93	0.65	0.12	0.29	0.35	0.27	0.85	0.33	1.57	0.00	0.00	0.20	0.21	0.53	2.73	0
83	unstocked	furuchatsunai	after stocking	154	30.70	0.95	5.57	2.30	1.35	5.94	1.25	3.18	3.28	0.51	0.06	0.18	0.26	0.20	0.72	0.25	1.51	0.00	0.00	0.19	0.21	0.50	2.29	0
84	unstocked	furuchatsunai	after stocking	143	31.50	0.58	5.89	2.05	1.31	6.28	0.99	3.00	4.76	0.65	0.05	0.19	0.20	0.21	0.62	0.32	1.49	0.00	0.00	0.00	0.18	0.46	2.25	0
85	unstocked	furuchatsunai	after stocking	137	26.81	0.84	5.09	1.96	1.26	5.07	0.98	2.46	3.54	0.49	0.14	0.04	0.15	0.16	0.54	0.24	1.37	0.02	0.00	0.00	0.10	0.32	2.02	0
86	unstocked	furuchatsunai	after stocking	122	44.19	1.08	9.12	2.75	1.88	9.57	1.27	4.44	7.15	0.86	0.32	0.05	0.28	0.25	0.57	0.41	1.59	0.05	0.00	0.00	0.03	0.45	2.06	0
mean				155	37.68	0.76	7.13	2.74	1.69	7.95	1.29	3.79	4.95	0.68	0.13	0.20	0.28	0.24	0.74	0.33	1.54	0.01	0.00	0.11	0.16	0.49	2.49	0
SD				22	7.83	0.20	1.64	0.73	0.36	2.14	0.25	0.89	1.32	0.14	0.10	0.12	0.08	0.06	0.17	0.07	0.10	0.02	0.00	0.11	0.07	0.12	0.48	0
mean (total)				171	35.69	0.57	6.79	2.51	1.57	7.62	1.13	3.42	4.94	0.61	0.11	0.18	0.25	0.21	0.66	0.30	1.50	0.01	0.00	0.07	0.25	0.44	2.56	0
SD (total)				29	8.03	0.23	1.73	0.89	0.34	1.93	0.39	0.74	1.70	0.23	0.09	0.10	0.06	0.04	0.12	0.08	0.40	0.02	0.00	0.09	0.15	0.22	0.31	0
				pearson's r	-0.40	**-0.63**	**-0.43**	-0.39	-0.30	-0.30	**-0.45**	-0.30	**-0.51**	**-0.57**	**-0.62**	**0.51**	0.24	-0.04	**0.43**	**-0.48**	**-0.52**	-0.19		0.34	0.26	0.32	**0.60**	

Supplementary table S1 (continued). Fatty acid content (mg/g-muscle-tissue) and fork length (mm) of experimental charr. Pearson's r values show correlations between fork length and fatty acid contents for each stream type and study period (bold text indicates significant correlations at $p < 0.05$). "no. of masu salmon fry" indicates the number of masu salmon fry preyed upon by each charr. All preyed-upon masu salmon fry were regarded as stocked hatchery fish. The study period "just after stocking" refers to 31 May, which was 4 days after stocking in Uchiyama and Matsumura and 6 days after stocking for the headstream side of the Mena River. Fatty acid analyses were not conducted for fish sampled just after stocking.

charr ID	stream type	sampling site	study period	fork length	**TFA**	14:0	16:0	16:1 n7	18:0	18:1 n9	18:1 n7	18:2 n6	18:3 n3	18:4 n3	20:1 n9	20:1 n7	20:2 n6	20:3 n6	20:4 n6	20:4 n3	**20:5n (EPA)**	22:1 n11	22:1 n9	22:4 n6	22:5 n6	22:5 n3	**22:6n (DHA)**	no. of masu salmon fry
87	stocked	uchiyama	just after stocking	181	-	-	-	-	-	-	-	-	-	-	-	-	-	-	-	-	-	-	-	-	-	-	-	5
88	stocked	uchiyama	just after stocking	141	-	-	-	-	-	-	-	-	-	-	-	-	-	-	-	-	-	-	-	-	-	-	-	10
89	stocked	uchiyama	just after stocking	142	-	-	-	-	-	-	-	-	-	-	-	-	-	-	-	-	-	-	-	-	-	-	-	4
90	stocked	uchiyama	just after stocking	136	-	-	-	-	-	-	-	-	-	-	-	-	-	-	-	-	-	-	-	-	-	-	-	6
91	stocked	uchiyama	just after stocking	110	-	-	-	-	-	-	-	-	-	-	-	-	-	-	-	-	-	-	-	-	-	-	-	1
92	stocked	uchiyama	just after stocking	102	-	-	-	-	-	-	-	-	-	-	-	-	-	-	-	-	-	-	-	-	-	-	-	0
93	stocked	uchiyama	just after stocking	95	-	-	-	-	-	-	-	-	-	-	-	-	-	-	-	-	-	-	-	-	-	-	-	0
mean				130	-	-	-	-	-	-	-	-	-	-	-	-	-	-	-	-	-	-	-	-	-	-	-	4
SD				30	-	-	-	-	-	-	-	-	-	-	-	-	-	-	-	-	-	-	-	-	-	-	-	4
94	stocked	matsumura	just after stocking	216	-	-	-	-	-	-	-	-	-	-	-	-	-	-	-	-	-	-	-	-	-	-	-	5
95	stocked	matsumura	just after stocking	203	-	-	-	-	-	-	-	-	-	-	-	-	-	-	-	-	-	-	-	-	-	-	-	9
96	stocked	matsumura	just after stocking	175	-	-	-	-	-	-	-	-	-	-	-	-	-	-	-	-	-	-	-	-	-	-	-	10
97	stocked	matsumura	just after stocking	159	-	-	-	-	-	-	-	-	-	-	-	-	-	-	-	-	-	-	-	-	-	-	-	17
98	stocked	matsumura	just after stocking	153	-	-	-	-	-	-	-	-	-	-	-	-	-	-	-	-	-	-	-	-	-	-	-	15
99	stocked	matsumura	just after stocking	141	-	-	-	-	-	-	-	-	-	-	-	-	-	-	-	-	-	-	-	-	-	-	-	3
100	stocked	matsumura	just after stocking	133	-	-	-	-	-	-	-	-	-	-	-	-	-	-	-	-	-	-	-	-	-	-	-	7
101	stocked	matsumura	just after stocking	138	-	-	-	-	-	-	-	-	-	-	-	-	-	-	-	-	-	-	-	-	-	-	-	2
102	stocked	matsumura	just after stocking	137	-	-	-	-	-	-	-	-	-	-	-	-	-	-	-	-	-	-	-	-	-	-	-	1
103	stocked	matsumura	just after stocking	134	-	-	-	-	-	-	-	-	-	-	-	-	-	-	-	-	-	-	-	-	-	-	-	0
104	stocked	matsumura	just after stocking	125	-	-	-	-	-	-	-	-	-	-	-	-	-	-	-	-	-	-	-	-	-	-	-	0
105	stocked	matsumura	just after stocking	122	-	-	-	-	-	-	-	-	-	-	-	-	-	-	-	-	-	-	-	-	-	-	-	2
106	stocked	matsumura	just after stocking	110	-	-	-	-	-	-	-	-	-	-	-	-	-	-	-	-	-	-	-	-	-	-	-	0
107	stocked	matsumura	just after stocking	119	-	-	-	-	-	-	-	-	-	-	-	-	-	-	-	-	-	-	-	-	-	-	-	0
mean				148	-	-	-	-	-	-	-	-	-	-	-	-	-	-	-	-	-	-	-	-	-	-	-	5
SD				31	-	-	-	-	-	-	-	-	-	-	-	-	-	-	-	-	-	-	-	-	-	-	-	6
108	stocked	kamimena	just after stocking	196	-	-	-	-	-	-	-	-	-	-	-	-	-	-	-	-	-	-	-	-	-	-	-	6
109	stocked	kamimena	just after stocking	188	-	-	-	-	-	-	-	-	-	-	-	-	-	-	-	-	-	-	-	-	-	-	-	0
110	stocked	kamimena	just after stocking	172	-	-	-	-	-	-	-	-	-	-	-	-	-	-	-	-	-	-	-	-	-	-	-	0
111	stocked	kamimena	just after stocking	171	-	-	-	-	-	-	-	-	-	-	-	-	-	-	-	-	-	-	-	-	-	-	-	4
112	stocked	kamimena	just after stocking	167	-	-	-	-	-	-	-	-	-	-	-	-	-	-	-	-	-	-	-	-	-	-	-	8
113	stocked	kamimena	just after stocking	148	-	-	-	-	-	-	-	-	-	-	-	-	-	-	-	-	-	-	-	-	-	-	-	3
114	stocked	kamimena	just after stocking	157	-	-	-	-	-	-	-	-	-	-	-	-	-	-	-	-	-	-	-	-	-	-	-	5
115	stocked	kamimena	just after stocking	148	-	-	-	-	-	-	-	-	-	-	-	-	-	-	-	-	-	-	-	-	-	-	-	4
116	stocked	kamimena	just after stocking	130	-	-	-	-	-	-	-	-	-	-	-	-	-	-	-	-	-	-	-	-	-	-	-	2
117	stocked	kamimena	just after stocking	128	-	-	-	-	-	-	-	-	-	-	-	-	-	-	-	-	-	-	-	-	-	-	-	0
mean				161	-	-	-	-	-	-	-	-	-	-	-	-	-	-	-	-	-	-	-	-	-	-	-	3
SD				23	-	-	-	-	-	-	-	-	-	-	-	-	-	-	-	-	-	-	-	-	-	-	-	3
mean (total)				148	-	-	-	-	-	-	-	-	-	-	-	-	-	-	-	-	-	-	-	-	-	-	-	4
SD (total)				30	-	-	-	-	-	-	-	-	-	-	-	-	-	-	-	-	-	-	-	-	-	-	-	4
				pearson's r																								

Supplementary table S1 (continued). Fatty acid content (mg/g-muscle-tissue) and fork length (mm) of experimental charr. Pearson's r values show correlations between fork length and fatty acid contents for each stream type and study period (bold text indicates significant correlations at $p < 0.05$). "no. of masu salmon fry" indicates the number of masu salmon fry preyed upon by each charr. All preyed-upon masu salmon fry were regarded as stocked hatchery fish. The study period "just after stocking" refers to 31 May, which was 4 days after stocking in Uchiyama and Matsumura and 6 days after stocking for the headstream side of the Mena River. Fatty acid analyses were not conducted for fish sampled just after stocking.

charr ID	stream type	sampling site	study period	fork length	**TFA**	14:0	16:0	16:1 n7	18:0	18:1 n9	18:1 n7	18:2 n6	18:3 n3	18:4 n3	20:1 n9	20:1 n7	20:2 n6	20:3 n6	20:4 n6	20:4 n3	**20:5n (EPA)**	22:1 n11	22:1 n9	22:4 n6	22:5 n6	22:5 n3	**22:6n (DHA)**	no. of masu salmon fry
118	unstocked	yuyanosawa	just after stocking	229	-	-	-	-	-	-	-	-	-	-	-	-	-	-	-	-	-	-	-	-	-	-	-	0
119	unstocked	yuyanosawa	just after stocking	189	-	-	-	-	-	-	-	-	-	-	-	-	-	-	-	-	-	-	-	-	-	-	-	0
120	unstocked	yuyanosawa	just after stocking	161	-	-	-	-	-	-	-	-	-	-	-	-	-	-	-	-	-	-	-	-	-	-	-	0
121	unstocked	yuyanosawa	just after stocking	153	-	-	-	-	-	-	-	-	-	-	-	-	-	-	-	-	-	-	-	-	-	-	-	0
122	unstocked	yuyanosawa	just after stocking	146	-	-	-	-	-	-	-	-	-	-	-	-	-	-	-	-	-	-	-	-	-	-	-	0
123	unstocked	yuyanosawa	just after stocking	142	-	-	-	-	-	-	-	-	-	-	-	-	-	-	-	-	-	-	-	-	-	-	-	0
124	unstocked	yuyanosawa	just after stocking	133	-	-	-	-	-	-	-	-	-	-	-	-	-	-	-	-	-	-	-	-	-	-	-	0
125	unstocked	yuyanosawa	just after stocking	126	-	-	-	-	-	-	-	-	-	-	-	-	-	-	-	-	-	-	-	-	-	-	-	0
126	unstocked	yuyanosawa	just after stocking	111	-	-	-	-	-	-	-	-	-	-	-	-	-	-	-	-	-	-	-	-	-	-	-	0
127	unstocked	yuyanosawa	just after stocking	107	-	-	-	-	-	-	-	-	-	-	-	-	-	-	-	-	-	-	-	-	-	-	-	0
mean				150	-	-	-	-	-	-	-	-	-	-	-	-	-	-	-	-	-	-	-	-	-	-	-	0
SD				37	-	-	-	-	-	-	-	-	-	-	-	-	-	-	-	-	-	-	-	-	-	-	-	0
128	unstocked	kayanuma	just after stocking	207	-	-	-	-	-	-	-	-	-	-	-	-	-	-	-	-	-	-	-	-	-	-	-	0
129	unstocked	kayanuma	just after stocking	174	-	-	-	-	-	-	-	-	-	-	-	-	-	-	-	-	-	-	-	-	-	-	-	0
130	unstocked	kayanuma	just after stocking	148	-	-	-	-	-	-	-	-	-	-	-	-	-	-	-	-	-	-	-	-	-	-	-	0
131	unstocked	kayanuma	just after stocking	154	-	-	-	-	-	-	-	-	-	-	-	-	-	-	-	-	-	-	-	-	-	-	-	0
132	unstocked	kayanuma	just after stocking	148	-	-	-	-	-	-	-	-	-	-	-	-	-	-	-	-	-	-	-	-	-	-	-	0
133	unstocked	kayanuma	just after stocking	121	-	-	-	-	-	-	-	-	-	-	-	-	-	-	-	-	-	-	-	-	-	-	-	0
134	unstocked	kayanuma	just after stocking	129	-	-	-	-	-	-	-	-	-	-	-	-	-	-	-	-	-	-	-	-	-	-	-	0
135	unstocked	kayanuma	just after stocking	116	-	-	-	-	-	-	-	-	-	-	-	-	-	-	-	-	-	-	-	-	-	-	-	0
136	unstocked	kayanuma	just after stocking	115	-	-	-	-	-	-	-	-	-	-	-	-	-	-	-	-	-	-	-	-	-	-	-	0
137	unstocked	kayanuma	just after stocking	109	-	-	-	-	-	-	-	-	-	-	-	-	-	-	-	-	-	-	-	-	-	-	-	0
mean				142	-	-	-	-	-	-	-	-	-	-	-	-	-	-	-	-	-	-	-	-	-	-	-	0
SD				31	-	-	-	-	-	-	-	-	-	-	-	-	-	-	-	-	-	-	-	-	-	-	-	0
138	unstocked	furuchatsunai	just after stocking	256	-	-	-	-	-	-	-	-	-	-	-	-	-	-	-	-	-	-	-	-	-	-	-	0
139	unstocked	furuchatsunai	just after stocking	197	-	-	-	-	-	-	-	-	-	-	-	-	-	-	-	-	-	-	-	-	-	-	-	0
140	unstocked	furuchatsunai	just after stocking	198	-	-	-	-	-	-	-	-	-	-	-	-	-	-	-	-	-	-	-	-	-	-	-	0
141	unstocked	furuchatsunai	just after stocking	159	-	-	-	-	-	-	-	-	-	-	-	-	-	-	-	-	-	-	-	-	-	-	-	0
142	unstocked	furuchatsunai	just after stocking	127	-	-	-	-	-	-	-	-	-	-	-	-	-	-	-	-	-	-	-	-	-	-	-	0
143	unstocked	furuchatsunai	just after stocking	125	-	-	-	-	-	-	-	-	-	-	-	-	-	-	-	-	-	-	-	-	-	-	-	0
144	unstocked	furuchatsunai	just after stocking	118	-	-	-	-	-	-	-	-	-	-	-	-	-	-	-	-	-	-	-	-	-	-	-	0
145	unstocked	furuchatsunai	just after stocking	111	-	-	-	-	-	-	-	-	-	-	-	-	-	-	-	-	-	-	-	-	-	-	-	0
146	unstocked	furuchatsunai	just after stocking	115	-	-	-	-	-	-	-	-	-	-	-	-	-	-	-	-	-	-	-	-	-	-	-	0
147	unstocked	furuchatsunai	just after stocking	108	-	-	-	-	-	-	-	-	-	-	-	-	-	-	-	-	-	-	-	-	-	-	-	0
mean				151	-	-	-	-	-	-	-	-	-	-	-	-	-	-	-	-	-	-	-	-	-	-	-	0
SD				50	-	-	-	-	-	-	-	-	-	-	-	-	-	-	-	-	-	-	-	-	-	-	-	0
mean (total)				148	-	-	-	-	-	-	-	-	-	-	-	-	-	-	-	-	-	-	-	-	-	-	-	0
SD (total)				39	-	-	-	-	-	-	-	-	-	-	-	-	-	-	-	-	-	-	-	-	-	-	-	0
				pearson's r																								

Supplementary Table S2. The results of linear mixed models testing the effects of study period, stream type, fork length and their interaction terms on the DHA, EPA and TFA contents of white-spotted charr.

	$df_{numerator}$	$df_{denominator}$	*F*	*p*
DHA				
Study period (SP)	1	81	61.15	<0.001
Stream type (ST)	1	81	4.762	0.032
Fork length (FL)	1	81	35.09	<0.001
SP×ST	1	81	47.51	<0.001
SP × FL	1	79	0.396	0.531
ST × FL	1	79	1.798	0.184
SP ×ST × FL	1	78	0.114	0.737
EPA				
Study period (SP)	1	77.17	51.68	<0.001
Stream type (ST)	1	3.995	6.698	0.061
Fork length (FL)	1	78.19	31.08	<0.001
SP×ST	1	77.03	28.42	<0.001
SP × FL	1	75.55	0.280	0.598
ST × FL	1	76.30	<0.001	0.993
SP ×ST × FL	1	74.54	0.260	0.612
TFA				
Study period (SP)	1	77.65	246.4	<0.001
Stream type (ST)	1	3.980	29.32	0.006
Fork length (FL)	1	80.53	1.632	0.205
SP×ST	1	77.11	32.99	<0.001
SP × FL	1	76.81	1.127	0.292
ST × FL	1	78.52	0.406	0.526
SP ×ST × FL	1	75.88	3.209	0.077

Table S3. The results of linear mixed models testing the effects of stream type, fork length and their interaction terms on the DHA, EPA and TFA contents of white-spotted charr before and after stocking.

	$df_{numerator}$	$df_{denominator}$	*F*	*p*
DHA				
Before stocking				
Stream type (ST)	1	3.709	6.027	0.075
Fork length (FL)	1	38.20	10.65	0.002
ST × FL	1	37.47	0.276	0.602
After stocking				
Stream type (ST)	1	41.00	37.52	<0.001
Fork length (FL)	1	41.00	21.13	<0.001
ST × FL	1	40.00	1.329	0.256
EPA				
Before stocking				
Stream type (ST)	1	4.005	22.20	0.009
Fork length (FL)	1	36.38	33.50	<0.001
ST × FL	1	35.51	0.109	0.744
After stocking				
Stream type (ST)	1	4.000	0.916	0.393
Fork length (FL)	1	38.09	10.68	0.002
ST × FL	1	37.11	0.003	0.959
TFA				
Before stocking				
Stream type (ST)	1	4.000	42.60	0.003
After stocking				
Stream type (ST)	1	3.998	3.478	0.136

Footnote: FL was not considered in TFA because a preliminary analysis revealed that FL did not correlate TFA (Table S2; Figure 2).

Supplementary table S4. Formulae of regression lines shown in Figure 2. y, $\log_{10}$ DHA or EPA; x, $\log_{10}$ fork length.

Fatty acid	Study period	Stream type	Estimated formula
DHA	Before stocking	Stocked	y=0.39x-0.55
		Unstocked	y=0.39x-0.49
	After stocking	Stocked	y=0.59x-0.80
		Unstocked	y=0.59x-0.91
EPA	Before stocking	Stocked	y=-1.05x+2.16
		Unstocked	y=-1.05x+2.45
	After stocking	Stocked	y=-0.59x+1.37
		Unstocked	y=-0.59x+1.47

Supplementary table S5.Fatty acid content (mg/g-muscle-tissue for fish, mg/g-whole-body for invertebrates) of stocked masu salmon and several aquatic invertebrates. The study period “just after stocking” refers to 31 May, which was 4 days after stocking in the Uchiyama stream. Some specimens of aquatic invertebrates were pooled because their body size was too small for analysis.

organisms	stream type	sampling site	study period	fork length	**TFA**	14:0	16:0	16:1 n7	18:0	18:1 n9	18:1 n7	18:2 n6	18:3 n3	18:4 n3	20:1 n9	20:1 n7	20:2 n6	20:3 n6	20:4 n6	20:4 n3	**20:5n (EPA)**	22:1 n11	22:1 n9	22:4 n6	22:5 n6	22:5 n3	**22:6n (DHA)**
stocked masu salmon	stocked	uchiyama	just after stocking	42.5	13.10	0.26	2.39	0.74	0.62	1.97	0.55	1.07	0.23	0.11	0.12	0.11	0.06	0.05	0.24	0.10	0.94	0.03	0.00	0.00	0.04	0.40	3.06
stocked masu salmon	stocked	uchiyama	just after stocking	46.5	22.51	0.55	4.17	1.40	0.96	4.06	0.81	2.78	0.40	0.17	0.31	0.31	0.10	0.10	0.34	0.15	1.20	0.07	0.00	0.00	0.08	0.46	4.10
stocked masu salmon	stocked	uchiyama	just after stocking	48.0	21.31	0.54	3.98	1.19	0.96	3.89	0.78	2.63	0.37	0.16	0.30	0.28	0.09	0.10	0.29	0.16	1.11	0.27	0.07	0.00	0.08	0.03	4.05
stocked masu salmon	stocked	uchiyama	just after stocking	47.5	19.48	0.45	3.45	0.94	0.87	3.49	0.71	2.52	0.33	0.13	0.28	0.27	0.10	0.01	0.31	0.13	1.07	0.25	0.06	0.00	0.08	0.37	3.67
stocked masu salmon	stocked	uchiyama	just after stocking	43.0	15.15	0.28	2.76	0.64	0.73	2.46	0.54	1.69	0.29	0.08	0.19	0.18	0.08	0.06	0.28	0.10	0.85	0.16	0.04	0.00	0.06	0.34	3.34
stocked masu salmon	stocked	uchiyama	just after stocking	48.0	25.88	0.66	4.54	1.71	1.03	4.69	1.06	3.10	0.57	0.25	0.34	0.33	0.11	0.02	0.36	0.21	1.72	0.31	0.07	0.00	0.09	0.60	4.11
stocked masu salmon	stocked	uchiyama	just after stocking	49.5	14.24	0.28	2.53	0.67	0.66	2.22	0.52	1.41	0.25	0.13	0.16	0.16	0.06	0.06	0.27	0.10	0.97	0.14	0.04	0.00	0.05	0.39	3.16
stocked masu salmon	stocked	uchiyama	just after stocking	47.5	15.93	0.31	2.82	0.71	0.74	2.66	0.55	1.74	0.23	0.09	0.19	0.20	0.09	0.05	0.28	0.11	1.00	0.17	0.04	0.00	0.05	0.38	3.52
ephemeroptera (3 individuals)	stocked	uchiyama	before stocking	-	51.81	2.82	14.11	8.80	1.50	4.08	4.54	0.84	3.04	3.49	0.00	0.08	0.00	0.07	0.34	0.16	7.95	0.00	0.00	0.00	0.00	0.00	0.00
ephemeroptera	stocked	uchiyama	before stocking	-	35.33	1.48	9.18	6.15	1.05	2.39	2.37	0.61	3.77	1.10	0.00	0.00	0.00	0.00	0.34	0.21	6.68	0.00	0.00	0.00	0.00	0.00	0.00
ephemeroptera (2 individuals)	unstocked	yuyanosawa	before stocking	-	44.17	0.98	12.04	8.11	1.10	3.13	4.72	0.53	5.29	0.89	0.00	0.00	0.00	0.00	0.29	0.08	6.99	0.00	0.00	0.00	0.00	0.00	0.00
plecoptera	unstocked	yuyanosawa	before stocking	-	99.48	0.88	17.75	11.81	2.86	22.11	8.54	3.66	12.74	0.29	0.19	0.07	0.05	0.07	1.45	0.17	16.38	0.10	0.00	0.00	0.06	0.08	0.21
tricoptera	unstocked	yuyanosawa	before stocking	-	34.23	2.65	7.14	0.18	1.86	4.58	1.42	4.63	3.36	0.15	0.00	0.00	0.00	0.00	0.43	0.11	7.61	0.00	0.00	0.00	0.00	0.00	0.11
